# The Effect of Gender and Sex Hormones on Cardiovascular Disease, Heart Failure, Diabetes, and Atrial Fibrillation in Sleep Apnea

**DOI:** 10.3389/fphys.2021.741896

**Published:** 2021-10-20

**Authors:** Philipp Hegner, Simon Lebek, Lars Siegfried Maier, Michael Arzt, Stefan Wagner

**Affiliations:** Department of Internal Medicine II, University Hospital Regensburg, Regensburg, Germany

**Keywords:** sleep apnea, sleep-disordered breathing, gender, atrial fibrillation, heart failure, cardiovascular disease, diabetes

## Abstract

Sleep apnea is a highly prevalent disorder with increasing impact on healthcare systems worldwide. Previous studies have been conducted primarily with male subjects, and prevalence and severity of sleep apnea in women are underestimated. Recent clinical and basic science evidence increasingly points to different mechanisms in men and women with sleep-disordered breathing (SDB). SDB is associated with a variety of comorbidities, including cardiovascular disease, heart failure, diabetes, and atrial fibrillation. In this review, we discuss sex-dependent mechanisms of SDB in select associated conditions to sharpen our clinical understanding of these sex-dependent inherent differences.

## Introduction

Sleep apnea is a highly prevalent disorder affecting up to one in four patients over the age of 65years and over one billion patients worldwide (Young et al., 1993; Benjafield et al., 2019). Sleep apnea has been shown to limit the quality of life of affected patients and is an independent risk factor for numerous comorbidities, including hypertension, cardiovascular disease, stroke, atrial fibrillation (AF), heart failure, and many others (Sahlin et al., 2008; Mokhlesi et al., 2016). In addition, patients with sleep-disordered breathing (SDB) have increased mortality (Lavie et al., 1995).

To date, research has focused primarily on male patients, and most basic science studies use male subjects. Although epidemiologic studies in community samples report a 2:1 male:female ratio for the prevalence of sleep apnea (Heinzer et al., 2015), women represent only 20–25% of patients in sleep apnea clinics (Marin et al., 2012). Recently, a large database study of over three million patients found an increased risk of several relevant comorbidities, such as type 2 diabetes, hypertension, cardiac arrhythmias, stroke, ischemic heart disease, depression, and congestive heart failure, in both men and women (Mokhlesi et al., 2016). Interestingly, diabetes and ischemic heart disease were more common in men, but hypertension and depression were more frequent in women with SDB. The prevalence of SDB and comorbidities is increased in postmenopausal women (Dancey et al., 2001) and in polycystic ovary syndrome (Vgontzas et al., 2001), suggesting the possibility that estrogen may play an important, possibly beneficial role.

Several mechanisms of AF development have been associated with SDB, most notably increased reactive oxygen species (ROS) production, increased diastolic sarcoplasmic reticulum (SR) Ca leak, increased late I_Na_ current (Lebek et al., 2020b), and reduced connexin 43 expression (Hegner et al., 2021). Interestingly, estrogen has recently been implicated in Na current regulation and Ca store handling (Firth et al., 2020).

Here, we discuss recent clinical and animal studies examining the influence of gender and sex hormones on mechanisms and comorbidities of sleep-disordered breathing.

## Influence of Gender on Manifestation of Heart Failure in SDB

Heart failure is an important comorbidity of SDB. Recently, it has been suggested that female SDB patients are more likely to develop heart failure with preserved ejection fraction (HFpEF) than heart failure with reduced ejection fraction (HFrEF; Lebek et al., 2021). In this study, 377 patients, approximately 84% of whom were men undergoing elective coronary artery bypass graft surgery were preoperatively tested for SDB. Interestingly, HFpEF was significantly more common overall in SDB patients compared to those without SDB (28 vs. 17%). This distribution was due to an increased frequency of HFpEF in female SDB patients (48% vs. only 25% in male). Echocardiographic characteristics of HFpEF were also significantly correlated with SDB severity; for example, female patients with SDB were significantly more likely to exhibit impaired diastolic left ventricular filling (echocardiographic E/eʹ) compared with men, and minimum oxygen saturation and time of oxygen saturation <90% were significantly correlated with E/eʹ.

In addition to HFpEF, HF with mildly reduced (HFmrEF) and reduced EF (HFrEF) constitute the other HF entities. Men are at least two times more likely to develop HFrEF compared with women (Lam et al., 2019). In a recent study analyzing 6,876 participants with chronic HFrEF treated according to guidelines, 79% were male (Arzt et al., 2016). Interestingly, SDB was present in 46% of patients in this HFrEF cohort, indicating that SDB is a frequent comorbidity in HF patients (Arzt et al., 2016; Ponikowski et al., 2016). However, similar to HFpEF, current knowledge about the mechanisms of sex differences is very limited (Ponikowski et al., 2016; Lam et al., 2019).

**Figure 1:**
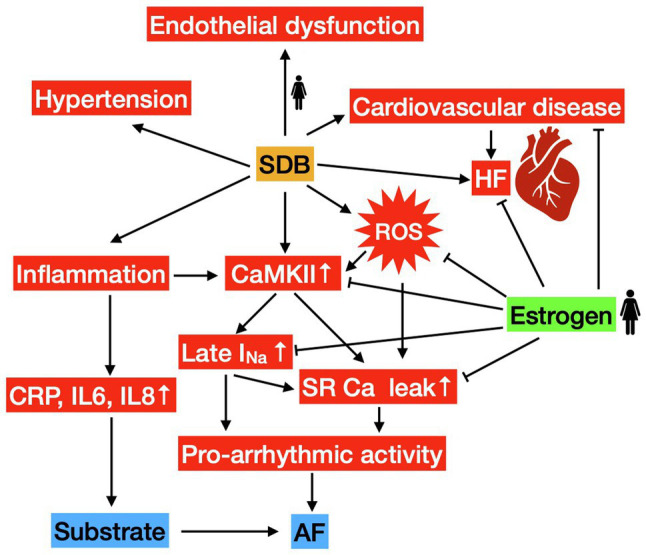
Gender-related mechanisms in sleep-disordered breathing (SDB). Stimulating pathways are depicted by arrows, protective/inhibitory effects of estrogen are denoted with a perpendicular line at the end. SDB leads to an increased risk of heart failure (HF), hypertension, endothelial dysfunction (in women), cardiovascular disease, inflammation, production of reactive oxygen species (ROS), and increased CaMKII activity. CaMKII-mediated SR Ca leak and Late I_Na_ facilitate pro-arrhythmic activity serving as trigger for atrial fibrillation (AF). Increased inflammation serves as part of the pro-arrhythmic substrate.

In this regard, diastolic SR Ca leak has been mechanistically linked to contractile dysfunction (Ai et al., 2005; Sossalla et al., 2010a), and our group has recently shown that Ca/Calmodulin-dependent protein kinase II (CaMKII)-induced SR Ca leak is increased in patients with SDB (Lebek et al., 2020b). However, there was no data for sex-dependent regulation in patients with SDB. SR Ca leak can be stimulated by CaMKII-dependent phosphorylation of SR Ca release channel ryanodine receptor 2 (RyR2). Interestingly, CaMKII is a cardiac stress enzyme that can be activated by ROS as seen in patients with SDB (Erickson et al., 2008; Lebek et al., 2020b). Estrogens have been shown to attenuate oxidative stress and decrease ROS generation in female rat cardiomyocytes (Lagranha et al., 2010). Moreover, expression and activity of CaMKII were increased in hearts of ovariectomized rats, which could be prevented by estrogen replacement (Ma et al., 2009). In contrast, testosterone was shown to increase activated CaMKII in rat cardiomyocytes (Duran et al., 2017). An overview of estrogen-dependent inhibition of detrimental pathways in SBD can be seen in Figure 1. Thus, decreased estrogen and increased testosterone levels may predispose men for oxidative activation of CaMKII, leading to increased diastolic SR Ca leak and risk of HFrEF development.

## Influence of Gender on Cardiovascular Disease in SDB

Large studies of SDB patients in the past were composed mainly of a male population and found an increased risk of cardiovascular disease and myocardial infarction (Hung et al., 1990). SDB includes obstructive sleep apnea (OSA), central sleep apnea (CSA), and mixed apnea, which describes respiratory effort initially and clear effort with obstruction at the end of the mixed apnea (Tietjens et al., 2019). More recently, women with OSA were also reported to be at higher risk of a wide range of comorbidities, including cardiovascular disease (OR 1.4; CVD), compared with control subjects, although CVD risk was slightly lower than in male OSA patients (Greenberg-Dotan et al., 2007). However, women display OSA more frequently than men (46 vs. 26% of SDB patients), who present mixed apnea and CSA more often (Arzt et al., 2017).

### Endothelial Dysfunction

One of the proposed mechanisms leading to the development of CVD is endothelial dysfunction and injury. Interestingly, Faulx et al. (2004) described marked endothelial dysfunction in female SDB patients. With increased apnea-hypopnea index (AHI), the flow-mediated dilation and peak blood flow were reduced after transient brachial artery cuff occlusion. Because this interaction was sex-specific for women, the authors conclude that women with SDB may be more susceptible to early SDB-related cardiovascular disease than men (Faulx et al., 2004). Endothelial dysfunction is also associated with thrombosis (Vita, 2011; Poredos and Jezovnik, 2018), and venous thrombosis and atherothrombosis demonstrate a shared pathophysiology (Piazza and Goldhaber, 2010). In a study of 82 patients with deep vein thrombosis (DVT) and/or pulmonary embolism (PE) and 82 controls, of which approximately half were female, Arzt et al. (2012) reported an increased risk for DVT and/or PE independent of established risk factors for thromboembolic events in SDB patients. Interestingly, this association was driven by a higher prevalence of SDB only in female DVT/PE patients (OR 4.14 compared to controls), but not in males.

### Hypertension

Hypertension is a well-established independent risk factor for the development of cardiovascular disease (Howard et al., 1999; Vasan et al., 2001; Wang et al., 2006). In addition, SDB is an independent risk factor for the development of hypertension (Peppard et al., 2000). Because frequent multiple comorbidities occur in SDB patients that are CVD risk factors in their own right, including obesity, diabetes, and metabolic syndrome, it is difficult to determine the individual effect of SDB on cardiovascular disease, but the risk for cardiovascular disease is thought to be mediated, at least in part, by elevated blood pressure (Leung and Bradley, 2001).

In contrast, the contribution of SDB to hypertension appears to be lower in women than in male patients (Hedner et al., 2006; Cano-Pumarega et al., 2017). While an increased OR for hypertension of 3.7 was reported for men with an AHI in the highest third of the population, no significant risk increase was observed in similar female patients (Hedner et al., 2006). This suggests that CVD risk is mediated differently in female SDB patients than in men.

### Stroke

Overall, age-specific stroke incidence is higher in men, but cumulative incidence in women exceeds due to higher life expectancy (Reeves et al., 2008). Although some studies report none or only minor gender differences in stroke incidence in SDB (Redline et al., 2010; Mokhlesi et al., 2016), there is evidence for a higher rate of stroke especially in younger women when compared with males (Chang et al., 2014). In a large database study by Chang et al. (2014), women under the age of 35 with SDB had the highest relative increase in stroke risk compared to controls, with an adjusted hazard ratio over four times higher than all other sex or age groups. These findings suggest that especially for younger women, SDB mediates a marked increase in stroke risk.

### Inflammation

Studies have shown increased levels of inflammation markers, such as CRP or IL6 in SDB, including a dose-dependent response (Shamsuzzaman et al., 2002; Motamedi et al., 2018). More recently, clinical evidence has demonstrated that there are gender differences in systemic inflammation in SDB. Gouveris et al. (2018) reported that CRP and fibrinogen were more strongly correlated with SDB severity in women than in men. The authors concluded that inflammation may be more severe in women with SDB.

In animal models of SDB, estrogen has been shown to play a role in mediating inflammation. Exposure of mice to chronic intermittent hypoxia (CIH) for 30days resulted in increased IL-6 and IL-8 levels, but no significant effect of CIH was observed in ovariectomized mice (Torres et al., 2014). Although this result might seem contrary to a protective effect of estrogen at first, the authors discuss various limitations and this study did not analyze clinical endpoints. In contrast, Lan et al. (2017) reported an increase in CIH-mediated aortic injury and oxidative stress in ovariectomized mice.

### Oxidative Stress

Estrogen, whose most biologically active form is estradiol (Yang et al., 1996), has been shown to possess antioxidant effects. In a clinical study of 19 healthy premenopausal women undergoing total hysterectomy with bilateral salpingo-oophorectomy, serum redox status, and antioxidant gene expression were assessed after surgery and estradiol replacement therapy (ERT; Bellanti et al., 2013). Compared with baseline, the authors reported a significant increase in ROS burden, indicated by oxidized (GSSG)/reduced (GSH) glutathione ratio. In addition, the expression of superoxide dismutase and glutathione peroxidase was reduced after surgery. In responders to ERT, these effects were reversible (Bellanti et al., 2013). In accordance, increased cardiovascular risk in postmenopausal women (Colditz et al., 1987; Wenger et al., 1993) has been attributed in part to lower estradiol levels and increased ROS (Strehlow et al., 2003).

On the one hand, progesterone has been shown to partially antagonize the anti-oxidative effects of estradiol *in vitro* (Wassmann et al., 2005). On the other hand, hormonal replacement therapy with progesterone analogs and estradiol was able to reduce apnea frequency in a small study of postmenopausal women (Wesstrom et al., 2005). Progesterone is known to act as a central respiratory stimulant (Bayliss et al., 1987). A randomized, controlled trial demonstrated higher nocturnal oxygen saturation and lower arterial carbon dioxide tension in SDB patients who received medroxyprogesterone acetate compared with placebo after discontinuation of CPAP therapy (Anttalainen et al., 2014). In addition, a case-control study of 144 women revealed lower serum progesterone levels in patients with sleep apnea (Lee et al., 2017). Overall, however, the effect of progesterone on cardiovascular disease in SDB has not been well studied to date.

In summary, estradiol and progesterone may be responsible for some of the differential manifestations of SDB in women compared with men.

## Influence of Gender on Diabetes Mellitus in SDB

Sleep-disordered breathing and diabetes are frequent comorbidities. Mainly, this includes type 2 diabetes, but also type 1 diabetes, and gestational diabetes in pregnant women (Pien et al., 2014; Mokhlesi et al., 2016; Reutrakul et al., 2016). Multiple effects of SDB on diabetes have been discussed, including worsening of glycemic control and increased insulin resistance (Reutrakul and Mokhlesi, 2017). Additionally, obesity is a common comorbidity both in SDB and diabetes patients, and some studies demonstrated higher BMI as a confounding variable for diabetes risk in SDB patients (Kent et al., 2014). However, diabetic patients suffer from peripheral and autonomic polyneuropathy more frequently (Banghoej et al., 2017), which in turn presents a risk factor for loss of upper airway patency and obstruction during sleep (Guilleminault et al., 1981).

Interestingly, a long-term follow-up study of a sleep clinic cohort demonstrated an apparent gender-dependent risk of new-onset diabetes in patients with SDB (Celen et al., 2010). Celen et al. (2010) report that when including age, BMI, and OSA in a multivariate logistic regression model, BMI remained the only significant predictor for diabetes risk in male patients, whereas OSA remained a significant predictor for diabetes in females. With an odds ratio of over 11, the authors indicate a markedly increased risk of new-onset diabetes in female SDB patients, whereas the higher incidence of diabetes in SDB males is rather mediated by an accompanying higher BMI. These results are in accordance with Valham et al. (2009), who reported an increased risk for diabetes in patients reporting snoring or witnessed sleep apneas in questionnaires, both symptoms of SDB. Interestingly, this association was limited to women of all ages as well as men under 55years, implying a possible gender-specific effect. On the other hand, some studies reported an association of SDB with diabetes only in men (Heinzer et al., 2018), and a higher prevalence of diabetes in male SDB patients overall without adjustment for previously stated clinical variables (Mokhlesi et al., 2016).

In addition to clinical studies, there is also increasing experimental evidence for sex-specific risk of diabetes in SDB. In a current animal study of mice undergoing intermittent hypoxia (IH) for 2weeks, impaired glucose tolerance was evident in female IH mice, but not males (Marcouiller et al., 2021). Furthermore, ovariectomy aggravated glucose intolerance, but this effect was reversible upon estrogen substitution therapy. The authors discuss possible mechanisms of this gender-dependent effect, including hormonal effects on pancreatic β cells and altered expression of metabolic genes (Marcouiller et al., 2021).

## Gender Differences in Atrial Fibrillation in SDB

Sleep-disordered breathing is associated with increased risk of arrhythmias (Monahan et al., 2009), AF (Gami et al., 2004, 2007), and recurrence of AF after pulmonary vein isolation therapy (Linz et al., 2018). In particular, diastolic SR Ca leak and increased Na influx play an important role in the development of AF and have both been associated with development of arrhythmias in SDB (Lebek et al., 2020b).

Interestingly, estrogen has recently been implicated in Na current regulation and Ca store handling (Firth et al., 2020). In a guinea pig HF model, Firth et al. (2020) describe an exacerbation of pressure overload-induced HF in ovariectomized animals. In addition to clinical endpoints, the authors report an increased sarcoplasmic reticulum (SR) Ca leak and reduced Ca transient amplitude as hallmarks of HF *in vitro*. Moreover, action potential duration (APD) and late I_Na_ current were increased in failing myocytes. Notably, SR Ca leak and late I_Na_ were further increased in ovariectomized animals, and this effect was reversible after estradiol substitution therapy (Firth et al., 2020). In addition to its role in HF, CaMKII has been associated with the development of arrhythmias and there are already several CaMKII inhibitors preclinically tested as antiarrhythmic drugs (Lebek et al., 2018; Neef et al., 2018). Mechanistic links to increased diastolic SR Ca leak and increased late I_Na_ have been established (Ai et al., 2005; Sossalla et al., 2010a; Wagner et al., 2011; Sag et al., 2014; Lebek et al., 2020b). Sex-hormone dependent CaMKII expression and activity have been described, as previously discussed, possibly allowing for some of the sex-specific variation in SR Ca Leak and late I_Na_ regulation (Ma et al., 2009; Duran et al., 2017). In addition, oxidative CaMKII activation due to differences in ROS may play a role. An overview of estrogen-dependent inhibition of pro-arrhythmogenic pathways in SBD can be seen in Figure 1.

As mentioned above, Lebek et al. (2020b) have associated increased production of ROS with SDB patients. In this study, interestingly, the severity of SDB was also significantly correlated with the magnitude of ROS production. In addition to oxidative CaMKII activation and the resulting increased SR Ca leak *via* RyR2 phosphorylation, direct oxidation of RyR2 has also been shown to enhance SR Ca leak (Terentyev et al., 2008). In this context, the anti-oxidative effects of estrogen may play a role, as discussed in more detail above (Strehlow et al., 2003).

Increased late I_Na_ and SR Ca leak can trigger arrhythmias by enhancing the frequency of early afterdepolarizations (EAD) and delayed afterdepolarizations (DAD) in the cardiac action potential (Neef et al., 2010; Sossalla et al., 2010b). EADs and DADs have been linked to onset of AF (Burashnikov and Antzelevitch, 2003; Chen and Tan, 2007) and occur more frequently in SDB patients (Lebek et al., 2020b). Intriguingly, the propensity for multicellular arrhythmias (premature atrial contractions, PACs) was also increased in atrial trabeculae of SDB patients (Lebek et al., 2020b). Gender-dependent differences in precursors of arrhythmias (EADs, DADs, and PACs) are not well studied to this date.

Recently, our group demonstrated that atrial expression of connexin 43, a cardiac gap junction protein responsible for conduction propagation, is reduced in SDB patients (Hegner et al., 2021). Moreover, reduced atrial connexin 43 expression was associated with an increased risk of developing atrial fibrillation in this study. Interestingly, the reduction in connexin expression was independent of multiple comorbidities, and in multivariate analysis, gender was not a relevant predictor.

In patients with paroxysmal and chronic AF, ion channel dysfunction and electrophysiological alterations, for example, APD shortening, contribute to the persistence of the arrhythmia (Voigt et al., 2014; Schmidt et al., 2015). For the sake of completeness, it should be noted that pro-arrhythmic mechanisms are partially different in paroxysmal, chronic, and postoperative AF (poAF). In the latter, changes in atrial myocyte electrophysiology were not observed in patients who developed poAF (Workman et al., 2006). Also, Fakuade et al. (2021) could recently demonstrate that impaired SR Ca reuptake contributes to the development of poAF rather than increased SR Ca leak. Therefore, in the development of poAF, an underlying pro-arrhythmogenic substrate, periprocedural-induced stress and inflammation, and specific alterations in myocyte Ca handling seem to play a role (Heijman et al., 2020; Nattel et al., 2020; Fakuade et al., 2021). Interestingly, patients with poAF are at eightfold increased risk of developing AF in the future, which softens the boundaries between different AF forms (Ahlsson et al., 2010).

While several studies have found an association between SDB and AF, there is limited information on differences between genders. In an analysis of the Framingham Heart Study cohort, Magnani et al. (2012) examined the risk of AF in a 10year follow-up. Of 1,809 women, 273 developed AF. There was no significant difference between the sexes in AF risk, regardless of whether the age at menopause was <45, 45–53, and >53years. However, the analyzed cohort consisted only of postmenopausal women over 60years of age; therefore, no analysis of pre- vs. postmenopausal women was performed. In addition, the prevalence of SDB has not been reported and was estimated to be as low as 9% in a similar cohort from Framingham Heart Study (Guidry et al., 2001).

Recently, a very large US nationwide health insurance database found a significant increase in several relevant comorbidities in women with SDB compared with controls, including higher incidence of arrhythmias and stroke (Mokhlesi et al., 2016). However, compared with matched male patients, there was little difference between genders.

## Conclusion

Due to the high prevalence and potentially severe comorbidities, such as hypertension, cardiovascular disease, stroke, atrial fibrillation, and heart failure, optimal treatment and understanding of the pathologies of SDB are essential. Because previous studies focused more on male subjects, the prevalence of SDB in women has historically been underestimated, and the impact of gender on SDB and its consequences is poorly understood. Women are known to develop equally relevant comorbidities of SDB. However, the underlying mechanisms likely differ.

In this review, we focused on the differential manifestation of HF, cardiovascular disease, diabetes, and arrhythmias in SDB. Increasing evidence suggests that women with SDB rather are more likely to develop HFpEF and men HFrEF. Knowledge on this topic is limited, and further studies investigating the underlying mechanisms are needed. Therefore, our group has recently published a novel mouse model of SDB by bulking agent-induced tongue enlargement (Lebek et al., 2020a). These mice not only show an increased frequency of spontaneous apneas and inspiratory flow limitations but also diastolic dysfunction, which offers a great opportunity for future investigation of gender-related effects in HFpEF and SDB.

In comparison, there are more studies, albeit still few, elucidating the impact of sex on cardiovascular disease in SDB. Here, differences in endothelial dysfunction, inflammation, production of ROS, and development of hypertension have been described (Figure 1). Despite the fact that the association between SDB and AF is well established, there is still limited information on the differences between men and women. Taken together, although current research does not suggest a significant difference in prevalence of AF by sex in SDB patients, sex hormones do play a role in pro-arrhythmic development. Several possible pro-arrhythmogenic mechanisms of AF have been investigated in both SDB patients and animal models. They include reduced connexin 43 expression, increased SR Ca leak, enhanced late I_Na_, and production of ROS. Recently, estrogen deficiency has been found to increase SR Ca leak and late I_Na_ in an animal study. However, further investigations in this field are needed to elucidate involved mechanisms and identify new therapeutic targets.

## Author Contributions

PH, SL, and SW contributed to manuscript drafting. PH, LM, MA, and SW contributed to manuscript review and editing. PH and SW revised the manuscript to its final form. All authors contributed to the article and approved the submitted version.

## Funding

LM was funded by the DFG grants MA 1982/5-1 and MA 1982/7-1. MA received grant support from the Else-Kroener Fresenius Foundation (2018_A159). SW was funded by DFG grants WA 2539/4-1, WA 2539/5-1, WA 2539/7-1, and WA 2539/8-1. SW and LM were also supported by the DFG SFB 1350 grant (project number 387509280, TPA6) and were funded by the ReForM C program of the Medical Faculty at the University of Regensburg. SL was supported by the ReForM A program of the Medical Faculty at the University of Regensburg.

## Conflict of Interest

MA received consulting fees from ResMed, Philips Respironics, Boehringer-Ingelheim, NRI, Novartis, Bayer, and Bresotec, and grant supports from ResMed as well as Foundation, all outside the submitted work.

The remaining authors declare that the research was conducted in the absence of any commercial or financial relationships that could be construed as a potential conflict of interest.

## Publisher’s Note

All claims expressed in this article are solely those of the authors and do not necessarily represent those of their affiliated organizations, or those of the publisher, the editors and the reviewers. Any product that may be evaluated in this article, or claim that may be made by its manufacturer, is not guaranteed or endorsed by the publisher.

## Abbreviations

AF: Atrial fibrillation
CaMKII: Ca/Calmodulin-dependent protein kinase II
CSA: Central sleep apnea
ERT: Estradiol replacement therapy
HF: Heart failure
HFpEF: Heart failure with preserved ejection fraction
HFmrEF: Heart failure with mildly reduced ejection fraction
HFrEF: Heart failure with reduced ejection fraction
OR: Odds ratio
OSA: Obstructive sleep apnea
ROS: Reactive oxygen species
SDB: Sleep-disordered breathing
